# Temporal Trends and Hospital Variation in Time-to-Antibiotics Among Veterans Hospitalized With Sepsis

**DOI:** 10.1001/jamanetworkopen.2021.23950

**Published:** 2021-09-07

**Authors:** Max T. Wayne, Sarah Seelye, Daniel Molling, Xiao Qing Wang, John P. Donnelly, Cainnear K. Hogan, Makoto M. Jones, Theodore J. Iwashyna, Vincent X. Liu, Hallie C. Prescott

**Affiliations:** 1 Division of Pulmonary and Critical Care Medicine, Department of Internal Medicine, University of Michigan, Ann Arbor; 2 VA Center for Clinical Management Research, Ann Arbor, Michigan; 3 Institute for Healthcare Policy & Innovation, University of Michigan, Ann Arbor; 4 Department of Learning Health Sciences, University of Michigan Medical School, Ann Arbor; 5 Salt Lake City VA Healthcare System, Salt Lake City, Utah; 6 Department of Medicine, University of Utah, Salt Lake City; 7 Division of Research, Kaiser Permanente Northern California, Oakland

## Abstract

**Importance:**

It is unclear whether antimicrobial timing for sepsis has changed outside of performance incentive initiatives.

**Objective:**

To examine temporal trends and variation in time-to-antibiotics for sepsis in the US Department of Veterans Affairs (VA) health care system.

**Design, Setting, and Participants:**

This observational cohort study included 130 VA hospitals from 2013 to 2018. Participants included all patients admitted to the hospital via the emergency department with sepsis from 2013 to 2018, using a definition adapted from the Centers for Disease Control and Prevention Adult Sepsis Event definition, which requires evidence of suspected infection, acute organ dysfunction, and systemic antimicrobial therapy within 12 hours of presentation. Data were analyzed from October 6, 2020, to July 1, 2021.

**Exposures:**

Time from presentation to antibiotic administration.

**Main Outcomes and Measures:**

The main outcome was differences in time-to-antibiotics across study periods, hospitals, and patient subgroups defined by presenting temperature and blood pressure. Temporal trends in time-to-antibiotics were measured overall and by subgroups. Hospital-level variation in time-to-antibiotics was quantified after adjusting for differences in patient characteristics using multilevel linear regression models.

**Results:**

A total of 111 385 hospitalizations for sepsis were identified, including 107 547 men (96.6%) men and 3838 women (3.4%) with a median (interquartile range [IQR]) age of 68 (62-77) years. A total of 7574 patients (6.8%) died in the hospital, and 13 855 patients (12.4%) died within 30 days. Median (IQR) time-to-antibiotics was 3.9 (2.4-6.5) hours but differed by presenting characteristics. Unadjusted median (IQR) time-to-antibiotics decreased over time, from 4.5 (2.7-7.1) hours during 2013 to 2014 to 3.5 (2.2-5.9) hours during 2017 to 2018 (*P* < .001). In multilevel models adjusted for patient characteristics, median time-to-antibiotics declined by 9.0 (95% CI, 8.8-9.2) minutes per calendar year. Temporal trends in time-to-antibiotics were similar across patient subgroups, but hospitals with faster baseline time-to-antibiotics had less change over time, with hospitals in the slowest tertile decreasing time-to-antibiotics by 16.6 minutes (23.1%) per year, while hospitals in the fastest tertile decreased time-to-antibiotics by 7.2 minutes (13.1%) per year. In the most recent years (2017-2018), median time-to-antibiotics ranged from 3.1 to 6.7 hours across hospitals (after adjustment for patient characteristics), 6.8% of variation in time-to-antibiotics was explained at the hospital level, and odds of receiving antibiotics within 3 hours increased by 65% (95% CI, 56%-77%) for the median patient if moving to a hospital with faster time-to-antibiotics.

**Conclusions and Relevance:**

This cohort study across nationwide VA hospitals found that time-to-antibiotics for sepsis has declined over time. However, there remains significant variability in time-to-antibiotics not explained by patient characteristics, suggesting potential unwarranted practice variation in sepsis treatment. Efforts to further accelerate time-to-antibiotics must be weighed against risks of overtreatment.

## Key Points

**Question:**

How has timing of antibiotics for sepsis changed over time in US Department of Veterans Affairs Hospitals?

**Findings:**

In this cohort of 111 385 veterans hospitalized with sepsis from 2013 to 2018, median time to antibiotics declined by 9 minutes per year. However, there was significant variation in time to antibiotics across hospitals, even after adjustment for patient characteristics.

**Meaning:**

These findings suggest that there is potential for performance improvement for sepsis hospitalizations, but efforts to further accelerate time-to-antibiotics must be weighed against the risks of overtreatment.

## Teaser

This cohort study examines temporal trends and variations in time-to-antibiotics for sepsis in the US Department of Veterans Affairs health care system.

## Introduction

Sepsis, life-threatening organ dysfunction secondary to infection, is a common, costly, and lethal syndrome, affecting more than 1.7 million patients annually in the United States.^1,2,3,4^ Sepsis quality improvement initiatives focus on rapid identification and administration of antimicrobials and fluids because there is higher quality evidence supporting these practices.^5,6,7,8^ Indeed, faster receipt of antimicrobial therapy has been associated with improved survival, particularly for patients who present with shock.^9,10,11^

Over the past 15 years, there has been growing attention to the importance of timely antimicrobial administration in sepsis. The 2016 Surviving Sepsis Campaign guidelines included a strong recommendation to administer antimicrobial therapy as soon as possible, ideally within 1 hour of recognition.^5^ Likewise, the US Center for Medicare and Medicaid Services’ SEP-1 performance measure and New York state’s “Rory’s Regulations” both incentivize timely antimicrobial administration.^10,12,13^ These and other sepsis performance improvement programs have resulted in faster administration of antimicrobials for their target populations, with associated improvements in inpatient sepsis survival over time.^14,15,16^ However, it is unclear whether antimicrobial timing for sepsis has changed outside of these formal performance incentive programs, and whether temporal trends in time-to-antibiotics have differed across hospitals or among patients who present with more obvious signs of sepsis.

In this study, we examined variation and temporal trends in time to antimicrobials for sepsis in 130 hospitals in the US Department of Veterans Affairs (VA) health care system. We hypothesized that time-to-antibiotics has decreased over time, that the magnitude of decrease has been uneven across patient subgroups and hospitals, and that time-to-antibiotics continues to vary across hospitals, even after adjustment for patient characteristics.

## Methods

The cohort study was deemed exempt from review with a waiver of informed consent by the VA Ann Arbor institutional review board. This study follows the Strengthening the Reporting of Observational Studies in Epidemiology (STROBE) reporting guideline for observational studies.

### Study Setting and Patient Cohort

The US VA health care system is a comprehensive health care system that operates a diverse set of hospitals.^17,18^ During the study period (2013-2018), the VA used a single electronic health record, with data archived to a central data repository.^18^ We identified all hospitalizations^19^ at nationwide VA hospitals admitted through the emergency department (ED) with community-acquired sepsis.

We defined community-acquired sepsis as in prior research,^20^ using a definition adapted from the Centers for Disease Control and Prevention (CDC) Adult Sepsis Event (ASE) definition.^21^ Specifically, we identified hospitalizations meeting the following criteria: admitted through the ED with 2 or more systemic inflammatory response (SIRS) criteria^10,22^; initiated on systemic antimicrobial therapy included in the CDC ASE definition^21^ within 12 hours of presentation^10^; continued on antibiotics for at least 4 consecutive days (or died prior to 4 days while receiving consecutive days of antibiotics); and had objective evidence of acute organ dysfunction^20^ within 48 hours of presentation. We further limited our cohort to hospitalizations at facilities with at least 15 eligible hospitalizations so that we could reliably measure variation across hospitals.^23,24,25,26^

For each hospitalization, we extracted demographics, comorbidities, vital signs, laboratory values, organ supports, length of stay, time of presentation to the ED, and mortality from the central data repository.^27^ Time-to-antibiotics was defined as the time from ED presentation to the time of initiation of the first antibiotic. Timing of antibiotic administration was determined via barcode medication administration (BCMA) data and physician order-entry records. For patients with an eligible antimicrobial ordered during their ED stay, we used BCMA where available. However, most VA EDs do not use BCMA, so we considered the time of antimicrobial administration to be the order-entry time plus 45 minutes. However, if patients were admitted to the hospital within 45 minutes of their antimicrobial order, then we considered time of transfer from ED-to-inpatient to be the time of first antimicrobial administration. Second, for patients without an antimicrobial order in the ED, we used BCMA to determine time of first antimicrobial administration (eAppendix in the Supplement). In sensitivity analyses, we considered several alternate approaches to defining time-to-antibiotics.

### Statistical Analysis

#### Temporal Trends in Time-to-Antibiotics for Sepsis

We present time-to-antibiotic results in minutes (when <60 minutes) and hours (when >60 minutes). We focus on median (rather than mean) and interquartile range (IQR) time-to-antibiotics because of skewed distribution.

To measure temporal trends in time-to-antibiotics for sepsis, we divided our study into early (2013-2014), middle (2015-2016), and late (2017-2018) periods. We examined the distribution of time-to-antibiotics by period, measured changes in the median time-to-antibiotics across periods, and tested for differences using a nonparametric test for trend. We compared the proportion of patients receiving antibiotics within 0 to 3, 0 to 6, and 0 to 9 hours over time. Finally, we fit multilevel linear regression models (hospitalizations nested within hospitals) estimating time-to-antibiotics, adjusting for patient characteristics (age, sex, 30 comorbid conditions identified using diagnosis codes from inpatient and outpatient encounters in the prior 1.5 years,^28,29,30^ individual SIRS criteria, lactate elevation, and 6 acute organ dysfunctions included in the CDC ASE definition^21^). We included a variable for calendar quarter and estimated the random slope for each hospital quarter to determine overall temporal trends in time-to-antibiotics. We calculated median time-to-antibiotics using fitted values for each patient that included the fixed-portion linear factor and contributions from the estimated random effects. In sensitivity analyses, we considered several alternate approaches to modeling. Additionally, in preliminary analyses, we assessed for a possible quadratic nonlinear association between calendar quarter and time-to-antibiotics, but we found no evidence of a nonlinear association during the study timeframe.

#### Variation in Time-to-Antibiotics and Temporal Trends by Patient Subgroups

We hypothesized that time-to-antibiotics and temporal trends in time-to-antibiotics may differ across hospitals and by patient subgroups. Specifically, we hypothesized that patients with more obvious signs of sepsis (eg, fever, hypotension) may have faster time-to-antibiotics and greater acceleration over time, that hospitals with faster baseline time-to-antibiotics in 2013 to 2014 would have less absolute change over time owing to floor effects, and that there would be clinically-significant variation in time-to-antibiotics across hospitals, even after adjustment for differences in patient characteristics.

To test these hypotheses, we assessed variation in time-to-antibiotics and temporal trends in time-to-antibiotics by hospitals, by tertiles of hospitals (grouped by baseline time-to-antibiotics), and by patient subgroups. Patient subgroups were defined by presenting temperature and blood pressure measured during the 25 hours surrounding ED presentation (24 hours pre-ED arrival to 1 hour post-ED arrival). Specifically, patients were classified as normothermic (≥36 °C and ≤38 °C), hypothermic (<36 °C), or hyperthermic (>38 °C), and as hypotensive (systolic blood pressure <90 mm Hg) or not hypotensive (systolic blood pressure ≥90 mm Hg). We used the most abnormal measurement during the 25-hour time-window of interest to classify patients and excluded (from this analysis only) 159 patients with both hypothermic and hyperthermic temperatures recorded. We assumed normal values when no measurements were recorded.

To quantify the variation in temporal trends across hospitals, we fit random-slope multilevel linear regression models with hospitalizations nested within hospitals, adjusting for patient characteristics as described above. We present variation in median time-to-antibiotics across hospitals in a caterpillar plot and assessed variation in slopes across hospitals. We measured the intraclass correlation (ICC) to quantify the proportion of variation in time-to-antibiotics attributable to the hospital where the patient was treated. The ICC measures the variation at the cluster level (ie, the hospital level) relative to total variation, where a value close to 0 indicates a low proportion of total variance explained at the hospital level and a value close to 1 indicates that nearly all variance in time-to-antibiotics is explained at the hospital level.^31^ We also calculated the median odds ratio (MOR) for receipt of antibiotics within 3 hours as a measure of variation between hospitals not explained by individual characteristics in the model.^32^ A MOR of 1.0 implies that odds of antibiotics within 3 hours is equivalent across hospitals; the larger the MOR, the more important hospital-level effects are in driving differences in time-to-antibiotics.

Data management and analysis were performed in SAS version 9.4 (SAS Institute) and Stata/MP version 16.1 (StataCorp). We considered 2-sided *P* < .05 to be significant. Data were analyzed from October 6, 2020, to July 1, 2021.

## Results

### Study Cohort

A total of 111 385 hospitalizations met criteria for community-acquired sepsis during the study period (eFigure 1 in the Supplement), including 107 547 men (96.6%) men and 3838 women (3.4%) with median (IQR) age of 68 (62-77) years (Table 1). Included patients had a median (IQR) of 2 (1-3) comorbid conditions. The most common acute organ dysfunctions were acute kidney dysfunction (68 191 patients [61.2%]), elevated lactate (53 136 patients [47.7%]), and thrombocytopenia (15 275 patients [13.7%]). A total 76 518 patients (68.7%) received their first dose of antibiotics in the ED, while 34 867 patients (31.3%) received their first dose after admission to the hospital. Over time, more patients received antibiotics in the ED (eTable 1 in the Supplement). Time-to-antibiotics was defined by order time plus 45 minutes in 51 086 patients (45.9%), ED-to-hospital transfer time in 16 943 patients (15.2%), and BCMA time in 43,356 patients (38.9%) (8489 patients [7.6%] in the ED; 34 867 patients [31.3%] on the wards). Among 8489 patients (7.6%) of patients with BCMA data in the ED, the median (IQR) time from order to administration was 23 (15-34) minutes. A total of 7574 patients (6.8%) died in the hospital, and 13 855 patients (12.4%) died within 30 days of discharge. In a multilevel model adjusted for patient characteristics, longer time-to-antibiotics was associated with higher in-hospital mortality (OR per 1-hour from ED presentation, 1.01 [95% CI, 1.00-1.02]; *P* = .02) and 30-day mortality (OR per 1-hour from ED presentation, 1.02 [95% CI, 1.01-1.02]; *P* < .001), consistent with prior studies.^9,10,11^

**Table 1.** Characteristics of Sepsis Hospitalizations in the Department of Veterans Affairs Health Care System From 2013 to 2018

Characterstic	No. (%) (N = 111 385)
Age, median (IQR), y	68 (62-77)
Sex	
Men	107 547 (96.6)
Women	3838 (3.4)
Race, %	
Black	21 828 (19.6)
White	80 598 (72.4)
Other^a^	8959 (8.0)
Comorbidities^b^	
Median (IQR), No.	2 (1-3)
Chronic pulmonary disease	52 353 (47.0)
Diabetes without complication	52 143 (46.8)
Kidney disease	38 741 (34.8)
Diabetes with complication	37 857 (34.0)
Congestive heart failure	37 202 (33.4)
Any cancer	27 854 (25.0)
Neurologic disease	21 408 (19.2)
Liver disease	20 445 (18.4)
Cancer with metastasis	9418 (8.5)
Acute organ dysfunction^c^	
Median (IQR), No.	1 (1-2)
Kidney	68 191 (61.2)
Elevated lactate	53 136 (47.7)
Hematologic	15 275 (13.7)
Hepatic	14 123 (12.7)
Shock	12 202 (11.0)
Respiratory	7632 (6.9)
ED LOS, median (IQR), h	4.6 (3.1-6.3)
LOS, median (IQR), d	7 (5-11)
In-hospital mortality	7574 (6.8)
30-d mortality	13 855 (12.4)

Abbreviations: ED, emergency department; IQR, interquartile range; LOS, length of stay.

^a^ Other race/ethnicity includes Asian, American Indian or Alaska Native, Native Hawaiian or other Pacific Islander, unknown, and declined to answer.

^b^ Comorbidities were identified from *International Classification of Diseases, Ninth Revision* (*ICD-9*) and *International Classification of Diseases, Tenth Revision* (*ICD-10*) codes in inpatient and outpatient encounters in the 1.5 years preceding hospitalization and classified according to Elixhauser criteria.

^c^ Acute organ dysfunction was identified from the electronic health record and defined as the following: elevated lactate required lactic acid greater than 18.02 mg/dL (to convert to millimoles per liter, multiply by 0.111); kidney dysfunction required a creatinine greater than 1.2 mg/dL (to convert to micromoles per liter, multiply by 76.25) and a 50% increase from baseline; shock required the receipt of intravenous vasopressors; hepatic dysfunction required total bilirubin >2.0 mg/dL (to convert to micromoles per liter, multiply by 17.104) and a 100% increase from baseline; hematologic dysfunction required platelet count, 100 cells/mL and a 50% decrease from baseline; and respiratory dysfunction required the receipt of invasive mechanical ventilation. Baseline organ function was measured via a 6-month look-back of laboratory data, as in prior studies.^20^

Patient characteristics by study time-period (early, middle, late) are presented in eTable 2 in the Supplement. Lactate elevation increased over time, likely reflective of greater testing, while all other acute organ functions declined (eTable 2 in the Supplement). In-hospital mortality declined from 2816 patients (8.1%) in the early period to 2330 patients (5.7%) in the late period, and 30-day mortality declined from 4876 patients (13.9%) in the early period to 4536 patients (11.2%) in the late period.

### Temporal Trend in Time-to-Antibiotics

**Figure 1.**
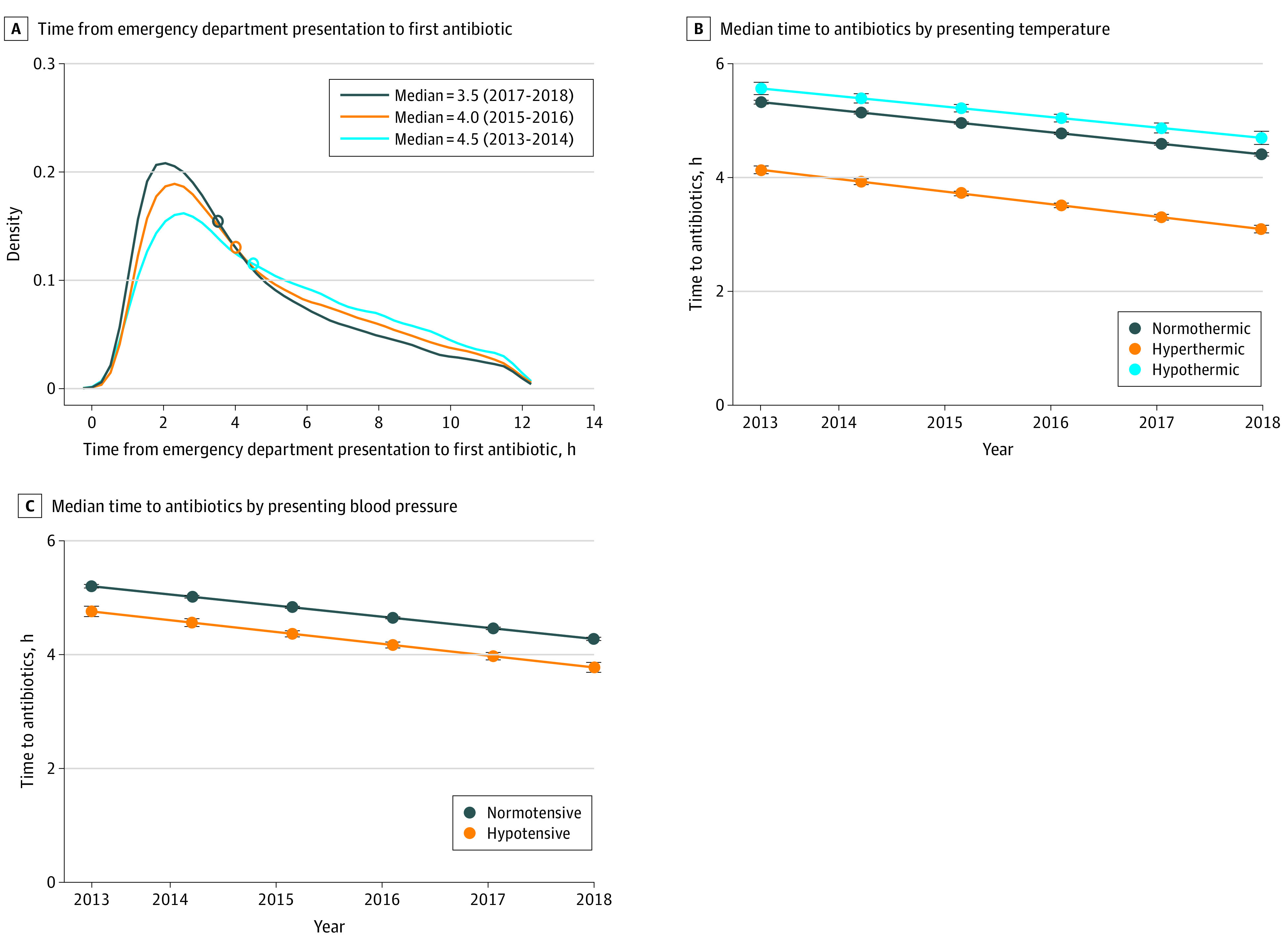
Time to First Antibiotic Administration by Study Period and by Patient Subgroups Over Time Patient subgroups were defined by presenting temperature and blood pressure measured during the 25 hours surrounding emergency department presentation (24 hours before arrival to 1 hour after arrival). Specifically, patients were classified as normothermic (≥36 °C and ≤38 °C), hypothermic (<36 °C), or hyperthermic (>38 °C) and as hypotensive (systolic blood pressure <90 mm Hg) or normotensive (≥90 mm Hg). Circles indicate median time-to-antibiotics.

The median (IQR) time-to-antibiotics during the study was 3.9 (2.4-6.5) hours. Median (IQR) time-to-antibiotics declined from 4.5 (2.7-7.1) hours in the early period to 3.5 (2.2-5.9) hours in late period, an absolute change of 54.6 minutes and a relative change of 22.2% (*P* < .001) (Figure 1A). The proportion of patients receiving antibiotics within 0 to 3, 0 to 6, and 0 to 9 hours increased for each comparison (eFigure 2 in the Supplement). After adjusting for patient characteristics, median time-to-antibiotics declined by 9.0 (95% CI, 8.8-9.2) minutes per calendar year. Annual change in time-to-antibiotics was similar across multiple sensitivity analyses using different definitions for time-to-antibiotics and different modeling approaches (Table 2).

**Table 2.** Annual Change in Time-to-Antibiotics in Primary and Sensitivity Analyses

Analysis	Change in time-to-antibiotic, min/y	
	Median (95% CI)	Mean (95% CI)
Primary analysis	9.03 (8.82-9.24)	9.04 (7.37-10.71)
Sensitivity analysis using different definitions for time-to-antibiotics for patients without BCMA data		
Order-entry time + 30 min	9.36 (9.14-9.58)	9.42 (7.72-11.13)
Order-entry time + 60 min	8.64 (8.43-8.85)	8.65 (7.01-10.29)
Order entry + random No. of min from 30-60	9.22 (8.99-9.46)	9.24 (7.56-10.93)
Time of first antimicrobial order	6.03 (5.88-6.19)	5.89 (4.55-7.22)
Sensitivity analyses using different modeling approaches		
Model 1^a^	9.03 (8.82-9.24)	9.04 (7.37-10.71)
Model 2^b^	9.72 (9.58-9.85)	9.68 (9.09-10.27)
Model 3^c^	10.60 (10.44-10.76)	10.52 (9.86-11.17)
Model 4^d^	9.98 (9.84-10.13)	9.87 (9.27-10.74)
Model 5^e^	9.17 (8.94-9.41)	8.40 (6.79-10.01)

Abbreviation: BCMA, bar-code medication administration.

^a^ Multilevel mixed-effects linear regression.

^b^ Single-level linear regression.

^c^ Single-level linear regression with log-transformed time-to-antibiotics.

^d^ Single-level generalized linear model, γ family, log link.

^e^ Multilevel mixed-effect generalized linear mode, γ family, log link.

### Variation in Time-to-Antibiotics by Patient Characteristics

Time-to-antibiotics varied markedly by patient characteristics. The distributions of time-to-antibiotics by patient subgroups (defined by presenting temperature and blood pressure) are shown in eFigure 3 in the Supplement. Median (IQR) time-to-antibiotics was 2.4 (1.6-4.1) hours for patients with fever and hypotension, 2.9 (1.8-4.7) hours for patients with fever and normal blood pressure, 3.6 (2.2-6.2) hours for patients without fever and with hypotension, and 4.3 (2.6-6.9) hours for patients without fever and with normal blood pressure (eFigure 3 in the Supplement). The association of individual patient characteristics with time-to-antibiotics are presented in eTable 3 in the Supplement. In fully adjusted models, lactate elevation (β = −36.9 [95% CI, −34.8 to −39.0] minutes; *P* < .001), abnormal white blood cell count (β = −30.9 [95% CI, −33.2 to −28.7] minutes; *P* < .001), abnormal temperature (β = −26.1 [95% CI, −24.1 to −28.0] minutes; *P* < .001), shock (β = −28.8 [95% CI, −32.0 to −25.6] minutes; *P* < .001), elevated respiratory rate (β = −21.2 [95% CI, −19.2 to −23.3] minutes; *P* < .001), and elevated heart rate (β = −17.9 [95% CI, −15.0 to −20.9] minutes; *P* < .001) were all associated with shorter time-to-antibiotics.

### Variation in Temporal Trends

**Figure 2.**
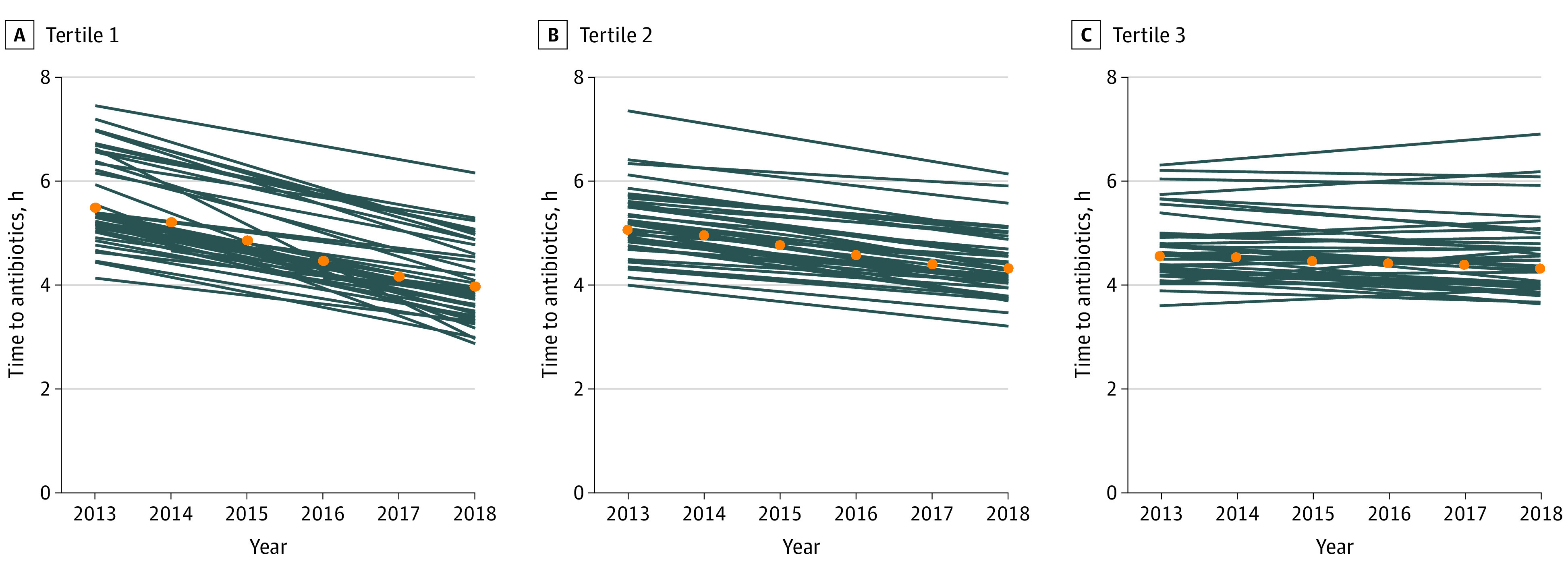
Temporal Trends in Time-to-Antibiotics Among Hospitals With the Largest, Middle, and Least Decline in Time-to-Antibiotics From 2013 to 2018 Tertile 1 declined by a median 19.1 minutes per year (44 hospitals); tertile 2 declined by a median of 10.1 minutes per year (43 hospitals); tertile 3 declined by a median 2.8 minutes per year (43 hospitals). Orange dots indicate median time-to-antibiotics per year; blue lines, time-to-antibiotics per individual hospital.

Time-to-antibiotics decreased over time in 112 hospitals (86.2%), but the magnitude of decline varied. Median time-to-antibiotics decreased by 19.1 (95% CI, 18.6-19.5) minutes per year in hospitals in the greatest tertile of decline, 10.1 (95% CI, 9.8-10.5) minutes per year in hospitals in the middle tertile of decline, and 2.8 (95% CI, 2.4-3.2) minutes per year for hospitals in the lowest tertile of decline, after adjustment for patient characteristics (Figure 2). Temporal trends likewise differed according to baseline time-to-antibiotics, decreasing by 16.6 minutes (23.1%) per year in hospitals with the slowest median time to antibiotics, 10.4 minutes (17.2%) per year in hospitals in the middle of the time-to-antibiotics tertile at baselines, and 7.2 minutes (13.1%) per year in hospitals with the fastest median time-to-antibiotics at baseline (*P* = .002 for the association of baseline tertile of time-to-antibiotics and tertile of change over time) (eTable 4 in the Supplement). Hospital characteristics were not associated with baseline time-to-antibiotics, but higher annual sepsis case-volume was associated with greater decline in time-to-antibiotics (eTable 5 and eTable 6 in the Supplement). While time-to-antibiotics varied by presenting temperature and blood pressure, declines over time were similar across these subgroups (Figure 1A; eTable 7 in the Supplement).

### Variation in Time-to-Antibiotics Across Hospitals in 2017 to 2018

**Figure 3.**
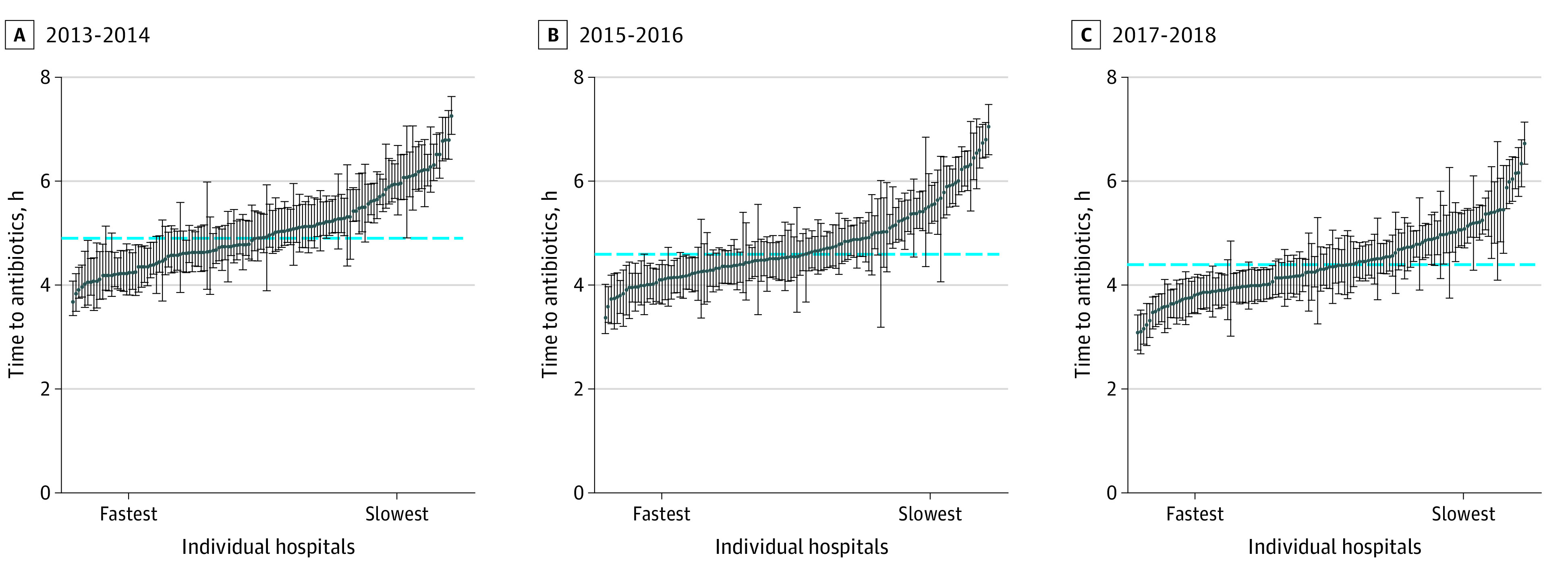
Variation in Median Time-to-Antibiotics by Hospital from 2013 to 2018 Individual hospitals are ordered from fastest to slowest time-to-antibiotics. The median time-to-antibiotics for each individual hospital is presented as a blue dot with corresponding 95% CIs (whiskers). The blue dotted line indicates the overall median time-to-antibiotic. The model intraclass correlation is 0.075 for 2013 to 2014, 0.081 for 2015 to 2016, and 0.068 for 2017 to 2018.

After adjusting for patient characteristics, median time-to-antibiotics varied by 118.2% across hospitals in 2017 to 2018—ranging from 3.1 to 6.7 hours (Figure 3). The ICC was 0.068, indicating that 6.8% of variation in time-to-antibiotics was explained at the hospital level. The MOR across hospitals for receipt of antibiotics within 3 hours of ED arrival was 1.65 (95% CI, 1.56-1.77), indicating a 65% increased odds of receiving antibiotics within 3 hours for the median patient if moving from a hospital with slower time-to-antibiotics to a hospital with faster time-to-antibiotics. The association between hospital and time-to-antibiotics was similar to the association of individual patient factors (eg, elevated lactate: OR, 1.61 [95% CI, 1.57-1.66]; shock: OR, 1.41 [95% CI, 1.35-1.47]). By contrast, in 2013 to 2014, median time-to-antibiotics varied by 95% (from 3.7 to 7.3 hours), the ICC was 7.5%, and the MOR for receipt of antibiotics within 3 hours was 1.73 (95% CI, 1.61-1.88).

## Discussion

In this nationwide cohort study of patients hospitalized with sepsis at 130 VA hospitals, we found that the median time-to-antibiotics for sepsis declined over time by 9 minutes per year. This trend of decreasing time-to-antibiotics was observed in virtually all hospitals and across all patient subgroups defined by presenting temperature and blood pressure. However, the magnitude of decrease varied across hospitals, and hospitals with faster baseline time-to-antibiotics experienced less change over time on either an absolute or relative scale.

Patient characteristics were associated with time-to-antibiotics. Most notably, lactate elevation, shock, and SIRS criteria (abnormal temperature, elevate heart rate, elevated respiratory rate, and abnormal white blood cell count) were associated with faster time-to-antibiotics, indicating that the urgency with which patient were administered antibiotics was associated with their clinical presentation. The variation in antibiotic timing observed across individual patients and the specific factors associated with faster delivery are generally consistent with a risk-based approach to antibiotic prescribing.^33,34^ However, time-to-antibiotics was faster for patients with fever and normal blood pressure than for patients without fever but with hypotension, suggesting that obvious signs of infection are a stronger trigger to prescribe antibiotics than shock or that competing treatments for patients with hypotension (eg, starting vasopressors) are prioritized before antibiotics, even though antibiotic delays are associated with greater mortality risk in patients with shock or hypotension.^9,10^

Although time-to-antibiotics differed according to patient characteristics, change over time was similar across patient subgroups. For example, patients with fever got antibiotics faster across the full study period, but the slope of change over time was similar to patients without fever.

While time-to-antibiotics declined overall and in nearly every hospital during the study, there remained more than 2-fold variation in median time-to-antibiotics in the most recent study years. This variation persisted after adjustment for granular patient characteristics, suggesting that sepsis practice patterns truly differ across hospitals. This may represent a potential opportunity for practice improvement going forward, but the benefits of further accelerating time-to-antibiotics must be balanced against the risk of driving antibiotic overuse in patients with noninfectious illness.^33^ It would be prudent to focus improvement efforts on patients with hypotension, for whom the risk of treatment delays is greatest.^33^

The trend of decreasing time-to-antibiotics observed in our study is similar to trends observed in other settings, including in intensive care units in Spain after implementation of a sepsis quality improvement (QI) initiative,^35^ hospitals participating in the international Surviving Sepsis Campaign QI program,^15^ and in New York state after implementation of statewide sepsis regulations.^14^ While a study by Liu et al^9^ found no change in antibiotic timing in 21 hospitals in the Kaiser Permanente Northern California Healthcare System after implementation of a regional sepsis QI project, the short baseline time-to-antibiotics and study design choices (eg, restricting the cohort to patients who received antibiotics within 6 hours, as opposed to 12 hours) may have limited the opportunity and ability to detect change.

The magnitude of change observed in our study (9 minutes per year) is greater than reported in all but 1 prior study,^35^ but our study population was less severely ill (hospital mortality rate, 6.8% vs 10% to 35% across prior studies^9,10,14,15,16^). The corresponding longer baseline time-to-antibiotics in our cohort may have provided a greater opportunity for decrease over time. Notably, however, prior studies were designed to assess changes in sepsis practice patterns after specific interventions, while ours was designed to test the change over time during a period of heightened national attention to sepsis, but not a specific sepsis intervention or quality improvement initiative. Indeed, the VA’s Nationwide Virtual Breakthrough Series^36,37^ on early identification and rapid treatment of sepsis in the emergency department occurred shortly after the study period (April to September 2019).

The variation in time-to-antibiotics across hospitals in our study is generally consistent with prior studies, although differences in study design and measurement (eg, measuring baseline as ED presentation vs when meeting sepsis criteria) preclude direct comparisons. In New York state, after implementation of Rory’s Regulations, the adjusted proportion of patients receiving antibiotics within 3 hours ranged from 60% to nearly 100% across hospitals.^10^ However, the slope of change over time varied across hospitals in this initiative, resulting in a growing disparity in time-to-antibiotics by race as hospitals treating higher proportions of Black patients experienced less decrease over time.^38^ We likewise found varying slopes of change across hospitals, which may be related to a ceiling effect whereby hospitals with the fastest time-to-antibiotics at the start of our study period had the least amount of change over time.

### Limitations

Our study has some limitations. First, there is no perfect method for identifying sepsis or defining “time zero.” We identified sepsis using the CDC surveillance definition because it identifies a stable population over time.^21^ Alternative methods, such as diagnostic codes, are subject to well-described temporal changes and varying sensitivities and specificities across hospitals,^39^ which would bias the measurement of temporal trends and cross-hospital comparisons of time-to-antibiotics.^1,40^ Our approach allowed for stable identification of sepsis over time and across hospitals and did not rely on physician or hospital identification of sepsis, which may result in underreporting. However, owing to increased ordering of lactate testing over time, this approach may have resulted in some differential ascertainment over time.^41^ Second, our definition required that patients received antibiotics within 12 hours. While the CDC ASE definition for community-acquired sepsis includes patients initiated on antibiotics within 48 hours of presentation, we used a 12-hour cutoff for consistency with prior studies of time-to-antibiotics.^10,42^ However, we tracked this exclusion, and only a small proportion of hospitalizations were excluded for having first antibiotic administration at more than 12 hours but less than 48 hours from ED presentation. Third, we are unable to determine the optimal time-to-antibiotics overall or for individual patients. While retrospective studies of patients with severe sepsis and septic shock have shown that faster time-to-antibiotics is associated with greater survival to hospital discharge, it is difficult to determine the presence of sepsis with certainty early in a patient’s presentation. Many patients prescribed antibiotics for potential sepsis turn out to have noninfectious causes of illness, and the benefits of early antibiotics must be balanced against the potential for overtreatment of patients who ultimately turn out to have noninfectious illness.^33^ Fourth, we used BCMA data to determine time of antibiotic administration where available, but for patients without these data, we estimated time-to-antibiotics based on physician order time. However, our assumption of 45 minutes from order to administration was conservative, and temporal trends were similar across several sensitivity analyses using alternate definitions for estimating time-to-antibiotics.

## Conclusions

In this large, national cohort study of veterans hospitalized with community-acquired sepsis, time-to-antibiotics declined by 9 minutes per year in the absence of a formal incentive program. However, there remains significant variation in time-to-antibiotics across hospitals that was not explained by differences in patient characteristics.

## Notes

Supplement. **eAppendix.** Calculating Time-to-Antibiotics **eFigure 1.** Patient Recruitment Flowchart **eTable 1.** Characteristics of Sepsis Hospitalizations by Location Where Patients Received First Dose of Antibiotics **eTable 2.** Characteristics of Sepsis Hospitalizations by Study Period **eFigure 2.** Time-to-Antibiotics Within 3, 6, and 9 Hours by Study Period **eFigure 3.** Time to First Antibiotic Administration by Presenting Temperature and Blood Pressure **eTable 3.** Association Between Patient Characteristics and Time to First Antibiotic Administration **eTable 4.** Association Between Baseline Time-to-Antibiotics and Change Over Time **eTable 5.** Hospital Characteristics by Baseline Time-to-Antibiotic Administration by Tertile **eTable 6.** Hospital Characteristics by Change in Time-to-Antibiotic Administration Over Time Tertile **eTable 7.** Time-to-Antibiotics Over Time by Sepsis Subgroups
eReferences
