# Supplementary material for: Temporal Trends and Hospital Variation in Time-to-Antibiotics Among Veterans Hospitalized With Sepsis
Source: JAMA Netw Open. 2021 Sep 7;4(9):e2123950. doi: 10.1001/jamanetworkopen.2021.23950 (PMC8424480; doi:10.1001/jamanetworkopen.2021.23950)
Supplement: Supplement. — eAppendix. Calculating Time-to-Antibiotics eFigure 1. Patient Recruitment Flowchart eTable 1. Characteristics of Sepsis Hospitalizations by Location Where Patients Received First Dose of Antibiotics eTable 2. Characteristics of Sepsis Hospitalizations by Study Period eFigure 2. Time-to-Antibiotics Within 3, 6, and 9 Hours by Study Period eFigure 3. Time to First Antibiotic Administration by Presenting Temperature and Blood Pressure eTable 3. Association Between Patient Characteristics and Time to First Antibiotic Administration eTable 4. Association Between Baseline Time-to-Antibiotics and Change Over Time eTable 5. Hospital Characteristics by Baseline Time-to-Antibiotic Administration by Tertile eTable 6. Hospital Characteristics by Change in Time-to-Antibiotic Administration Over Time Tertile eTable 7. Time-to-Antibiotics Over Time by Sepsis Subgroups eReferences [file jamanetwopen-e2123950-s001.pdf]

Supplemental Online Content

Wayne MT, Seelye S, Molling D, et al. Temporal trends and hospital variation in time-to-antibiotics among veterans hospitalized with sepsis. *JAMA Netw Open*. 2021;4(9):e2123950. doi:10.1001/jamanetworkopen.2021.23950

eAppendix. Calculating Time-to-Antibiotics

eFigure 1. Patient Recruitment Flowchart

eTable 1. Characteristics of Sepsis Hospitalizations by Location Where Patients Received First Dose of Antibiotics

eTable 2. Characteristics of Sepsis Hospitalizations by Study Period

eFigure 2. Time-to-Antibiotics Within 3, 6, and 9 Hours by Study Period

eFigure 3. Time to First Antibiotic Administration by Presenting Temperature and Blood Pressure

eTable 3. Association Between Patient Characteristics and Time to First Antibiotic Administration

eTable 4. Association Between Baseline Time-to-Antibiotics and Change Over Time

eTable 5. Hospital Characteristics by Baseline Time-to-Antibiotic Administration by Tertile

eTable 6. Hospital Characteristics by Change in Time-to-Antibiotic Administration Over Time Tertile

eTable 7. Time-to-Antibiotics Over Time by Sepsis Subgroups

eReferences

This supplemental material has been provided by the authors to give readers additional information about their work.

© 2021 Wayne MT et al. *JAMA Network Open.*

eAppendix. Calculating Time-to-Antibiotics

Time of presentation to the emergency department (ED) was extracted from the Emergency Department Information System (EDIS) domain within VA's Corporate Data Warehouse (CDW). We used the PatientArrivalDateTime variable from the Emergency Department Information System (EDIS) domain within the VA's Corporate Data Warehouse (CDW). This is the first timestamp available, and to the best of our knowledge represents when the patient arrived to the hospital and does not require triage to be complete. We used this same variable to define presentation time across all hospitals.

Time of first antibiotic administration after presentation to the ED was determined via bar-code medication administration data (where available) and physician order-entry records (otherwise). Specifically, we extracted systemic antibiotics considered as "qualifying antimicrobials" in the CDC's Adult Sepsis Event definition.[1]

We used a hierarchical procedure to determine time of first antibiotic administration. First, for patients with an eligible antimicrobial ordered during their ED stay, we used bar-code administration where available. However, the majority of VA EDs do not use bar-code administration, so we considered the time of antimicrobial administration to be the order-entry time plus 45 minutes (since time of antibiotic administration in the ED is not reliably recorded in the electronic health record and a prior study suggested this as a reasonable estimate of time from antibiotic order to administration[2]). However, if patients were admitted to the hospital within 45 minutes of their antimicrobial order, then we considered time of transfer from ED-to-inpatient to be the time of first antimicrobial administration (reasoning that antibiotic orders are generally executed prior to admission). Second, for patients without an antimicrobial order in the ED, we used bar-code medication administration records (within the CDW[3]) to determine time of first antimicrobial administration.

For our cohort, 45.9% of hospitalizations had their antibiotic administration time defined as order time plus 45 minutes, 15.2% as time of ED-to-inpatient transfer, and 38.9% (7.6% in the ED, 31.3% on the wards) as the first bar-code medication administration time.

For patients with BCMA data in the ED, median time from antibiotic order to antibiotic administration was 23 minutes (N=8,385; excludes 46 hospitalizations with no antibiotic order and 58 hospitalizations with administration prior to order time).

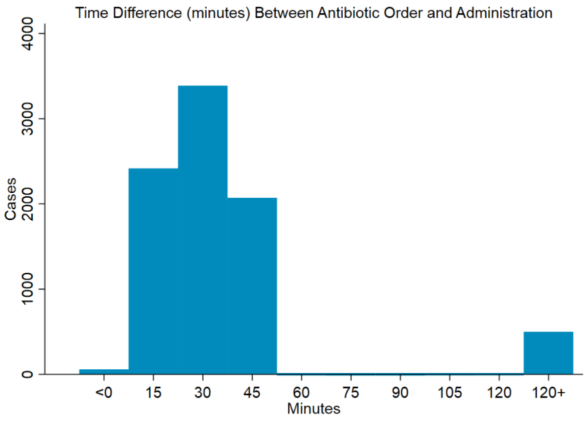

© 2021 Wayne MT et al. *JAMA Network Open.*

eFigure 1. Patient Recruitment Flowchart

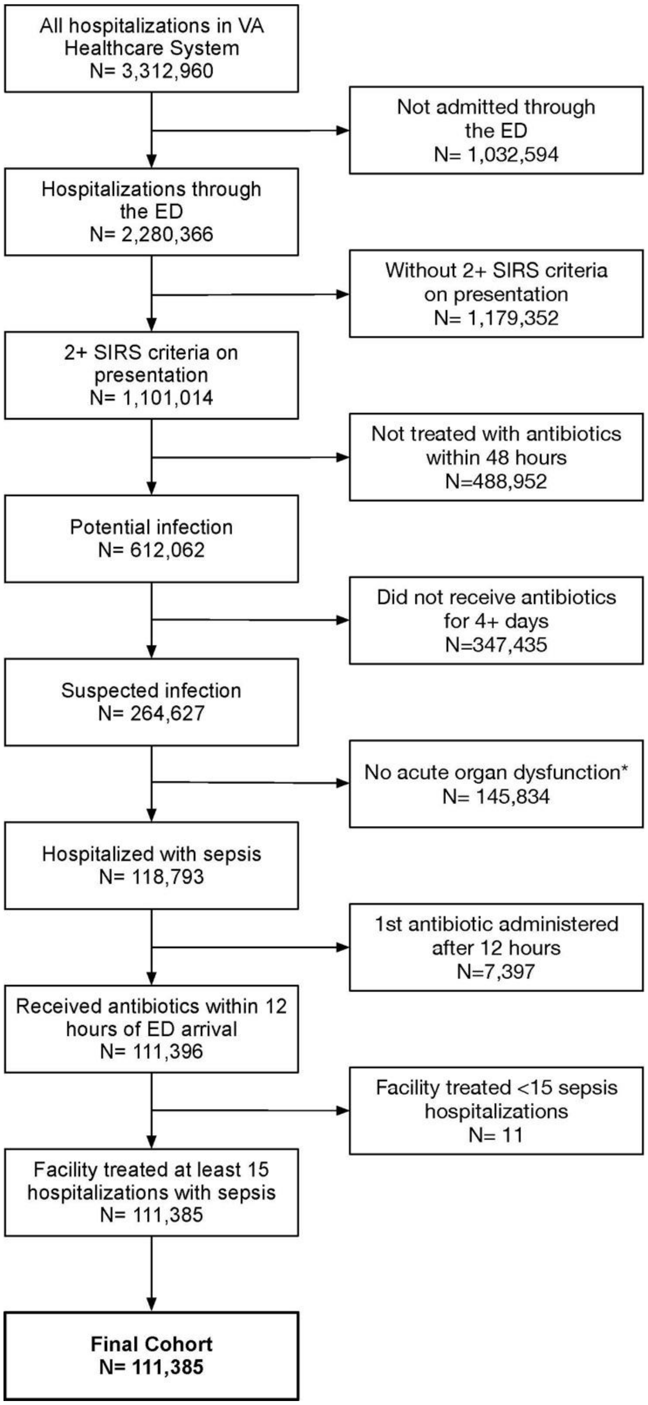

Figure 1 Legend: There were 3,312,960 hospitalizations from 2013 to 2018 in the VA Healthcare System, of which 111,385 hospitalizations met all study inclusion and exclusion criteria and were included in our study cohort.

© 2021 Wayne MT et al. *JAMA Network Open.*

eTable 1. Characteristics of Sepsis Hospitalizations by Location Where Patients Received First Dose of Antibiotics

	Received 1st antibiotic in ED (N=76,518)	Received 1st antibiotic following admission (N=34,867)
Age in years, median (IQR)	69 (62,77)	68 (62, 76)
Male, %	96.6	96.5
Race, %		
White	73.7	71.8
Black	18.5	20.1
Other	7.8	8.1
Comorbidities, %		
Diabetes without complication	46.6	47.4
Chronic pulmonary disease	45.3	50.8
Renal disease	33.8	36.9
Congestive heart failure	32.0	36.6
Any cancer	25.0	25.1
Diabetes with complication	34.5	32.8
Liver disease	17.1	21.1
Neurologic disease	19.7	18.3
Cancer with metastasis	8.4	8.6
Number of comorbidities, median (IQR)	2 (1,3)	2 (1,3)
Acute organ dysfunction, %		
Renal	59.8	64.3
Elevated lactate*	51.8	38.7
Hematologic	13.1	15.0
Hepatic	12.2	13.7
Shock	11.7	9.4
Respiratory	6.4	7.9
Number of acute organ dysfunctions, median (IQR)	1 (1,2)	1 (1,2)
Length of stay (days), median (IQR)	7 (5,11)	7 (5,11)
Length of stay in ED in hours, median (IQR)	5.0 (3.5,6.9)	3.7 (2.5, 5.2)
In-hospital mortality, %	6.6	7.2
30-day mortality, %	11.8	13.8
By time period, % receiving antibiotics in each location		
2013-2014	63.7	36.3
2015-2016	67.0	33.0
2017-2018	74.5	25.5

ED: emergency department; IQR: interquartile range

© 2021 Wayne MT et al. *JAMA Network Open.*

eTable 2. Characteristics of Sepsis Hospitalizations by Study Period

		Early<br>2013-2014<br>(n=34,985)	Middle<br>2015-2016<br>(n=35,731)	Late<br>2017-2018<br>(n=40,669)
Sepsis hospitalizations per hospital, median (IQR)		239<br>(120, 390)	244<br>(99, 408)	284<br>(126, 474)
Age in years, median (IQR)		67 (62,77)	68 (62,77)	69 (63,76)
Male, %		96.8	96.7	96.2
Race, %				
	White	72.6	72.8	71.8
	Black	19.0	19.3	20.4
	Other	8.4	7.9	7.9
Comorbidities, %				
	Diabetes without complication	45.7	49.6	45.4
	Chronic pulmonary disease	44.7	49.0	47.2
	Renal disease	32.7	35.5	36.0
	Congestive heart failure	30.3	34.2	35.4
	Any cancer	25.0	26.8	23.4
	Diabetes with complication	23.5	34.2	42.8
	Liver disease	16.3	19.0	19.5
	Neurologic disease	13.5	18.9	24.5
	Cancer with metastasis	8.3	9.0	8.1
Number of comorbidities, median (IQR)		2 (1,3)	2 (1,3)	2 (1,3)
Acute organ dysfunction, %				
	Renal	66.2	61.1	57.1
	Elevated lactate*	37.3	49.1	55.4
	Hematologic	15.4	13.5	12.4
	Hepatic	13.4	12.6	12.1
	Shock	11.3	10.7	10.9
	Respiratory	8.1	6.7	6.0
Number of acute organ dysfunctions, median (IQR)		1 (1,2)	1 (1,2)	1 (1,2)
Length of stay (days), median (IQR)		7 (5,11)	7 (5,11)	7 (4,10)
Length of stay in ED in hours, median (IQR)		4.3<br>(2.9, 6.1)	4.4<br>(3.1, 6.2)	4.8<br>(3.4, 6.6)
In-hospital mortality, %		8.1	6.8	5.7
30-day mortality, %		13.9	12.4	11.2

*The increasing proportion of patients with measured lactate elevation is reflective of increasing lactate measurement across the study years. The proportion of hospitalizations with a lactate measurement increased each year, from 56.5% in 2013 to 83.3% of hospitalizations in 2018.
ED: emergency department; IQR: interquartile range

© 2021 Wayne MT et al. *JAMA Network Open.*

eFigure 2. Time-to-Antibiotics Within 3, 6, and 9 Hours by Study Period

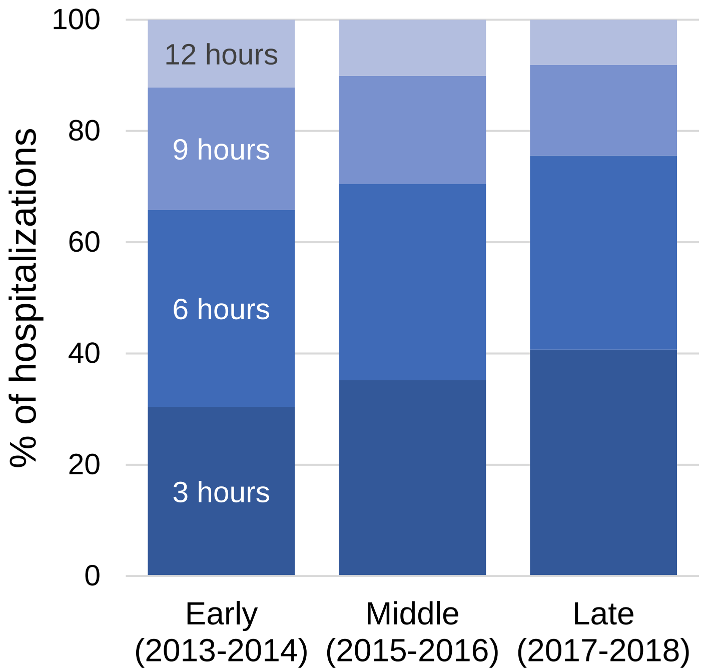

Figure 2 Legend: This stacked bar-graph shows the proportion of hospitalizations who received antibiotics within 3, 6, and 9 hours of emergency department (ED) presentation by study time-period. To be eligible for the cohort, all patients had to have received antibiotics within 12 hours of ED presentation. Median time-to-antibiotics declined over time, such that a greater proportion of hospitalizations received antibiotics within 3, 6, and 9 hours over time.

© 2021 Wayne MT et al. *JAMA Network Open.*

eFigure 3. Time to First Antibiotic Administration by Presenting Temperature and Blood Pressure

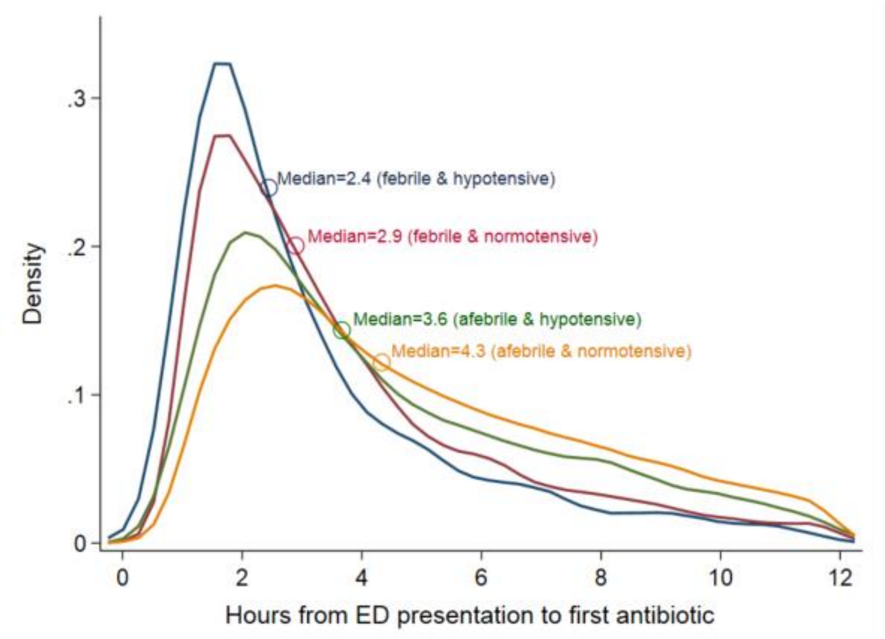

Figure 3 Legend: Kernel density plot showing time to first antibiotic administration from emergency department (ED) presentation by presenting temperature and blood. Patient subgroups were defined by presenting temperature and blood pressure measured during the 25 hours surrounding ED presentation (24 hours pre-ED arrival to 1 hour post-ED arrival). Specifically, patients were classified as: (1) normothermic (≥36°C and ≤38°Celsius); (2) hypothermic (<36°); or (3) hyperthermic (>38°); and as: (1) hypotensive (systolic blood pressure <90 mmHg) or (2) normotensive (≥90 mmHg). We used the most abnormal measurement during the 25-hour time-window of interest to classify patients, assumed normal values when no measurements were recorded, and excluded (from this analysis only) patients with both hypo- and hyperthermic temperatures recorded. Of the total cohort, 159 patients (0.1%) had temperature recordings that were both hypo- and hyperthermic and were therefore excluded from this analysis. Of the remaining patients, 76.5% were classified as normothermic (including 5.9% with no temperature recording during the time-window of interest), 17.2% were hyperthermic, and 6.3% were hypothermic; 89.7% were normotensive (including 4.2% with no blood pressure recording during the time-window of interest), and 10.3% were hypotensive.
°C: degrees Celsius; ED: emergency department; mmHg: millimeters of mercury

© 2021 Wayne MT et al. *JAMA Network Open.*

eTable 3. Association Between Patient Characteristics and Time to First Antibiotic Administration

Characteristics	Regression coefficient* (95% CI)	p-value
Age, per year	-0.1 (-0.2, -0.0)	0.03
Male	-16.2 (-214, -11.0)	<0.001
SIRS criteria on presentation		
Abnormal body temperature (>38°C or <36°C)	-26.1 (-28.046, -24.125)	<0.001
Respiratory rate >20 breaths per minute	-21.3 (-23.338, -19.175)	<0.001
Heart rate >90 beats per minute	-17.9 (-20.854, -14.963)	<0.001
White blood cell count >12,000 cells/µL or <4,000 cells/µL	-30.9 (-33.195, -28.678)	<0.001
Acute organ dysfunction		
Elevated lactate	-36.9 (-39.0, -34.8)	<0.001
Renal	-2.5 (-4.7, -0.375)	0.021
Shock	-28.8 (-32.0, -25.6)	<0.001
Hepatic	17.9 (14.9, 20.8)	<0.001
Hematologic	1.1 (-1.9, 4.1)	0.48
Respiratory	-11.1 (-15.1, -7.1)	<0.001
Comorbidities		
Cancer without metastasis	0.6 (-1.9, 3.1)	0.62
Cancer with metastasis	0.6 (-3., 4.4)	0.76
Chronic pulmonary disease	-5.9 (-7.6, -3.6)	<0.001
Congestive heart failure	4.1 (1.8, 6.4)	0.001
Diabetes without complication	3.2 (0.7, 5.6)	0.01
Diabetes with complication	-5.1 (-7.8, -2.5)	<0.001
Liver disease	14.9 (12.2, 17.6)	<0.001
Neurologic disease	-7.2 (-9.7, -4.7)	<0.001
Renal disease	3.4 (1.2, 5.6)	0.002
Hypertension with and without complication	1.6 (-0.9, 4.2)	0.20
Cardiac arrhythmia	-1.7 (-3.8, 0.3)	0.10
Valvular disease	1.1 (-2.0, 4.2)	0.50
Pulmonary circulation disorders	5.0 (1.9, 8.2)	<0.01
Peripheral vascular disease	-2.1 (-4.4, 0.2)	0.07
Paralysis	-16.6 (-21.1, -12.1)	<0.001
Peptic ulcer disease	7.9 (2.7, 13.1)	0.003
Hypothyroidism	-2.1 (-4.8, 0.5)	0.12
AIDS/HIV	6.6 (-1.0, 14.2)	0.09
Lymphoma	-1.1 (-5.9, 3.6)	0.64
Rheumatoid arthritis	-0.0 (-4.5, 4.5)	1.0
Coagulopathy	2.0 (-0.8, 4.8)	0.16
Obesity	0.2 (-2.1, 2.5)	0.88
Weight loss	-2.2 (-4.7, 0.4)	0.09
Fluid and electrolyte disorders	2.9 (0.8, 4.9)	0.01
Blood loss anemia	12.1 (7.1, 17.1)	<0.001
Deficiency anemia	-1.6 (-4.2, 1.0)	0.22
Alcohol abuse	10.5 (7.7, 13.3)	<0.001

© 2021 Wayne MT et al. *JAMA Network Open.*

	Drug abuse	1.0 (-2.3, 4.3)	0.56
	Psychoses	-4.6 (-8.2, -1.1)	0.01
	Depression	-2.9 (-5.0, -0.9)	<0.01

Wald chi-square=5257.33, p<0.001. The regression coefficients can be interpreted as the change in time-to-antibiotics associated with each characteristic.
AIDS: Acquired immunodeficiency syndrome; ℃: Celsius; ED: emergency department; HIV: human immunodeficiency virus; SIRS: severe inflammatory response syndrome

© 2021 Wayne MT et al. *JAMA Network Open.*

eTable 4. Association Between Baseline Time-to-Antibiotics and Change Over Time

Baseline time-to-antibiotics	Change over time		
	Tertile 1 (*i.e.,* least decline) N= 43 hospitals	Tertile 2 N= 43 hospitals	Tertile 3 (*i.e.,* greatest decline) N= 44 hospitals
Tertile 1 (*i.e., slowest baseline)* (N= 43 hospitals)	7 (5.4%)	13 (10.0%)	23 (17.7%)
Tertile 2 (N= 43 hospitals)	14 (10.8%)	15 (11.5%)	14 (10.8%)
Tertile 3 (*i.e.,* fastest baseline) (N= 44 hospitals)	22 (16.9%)	15 (11.5%)	7 (5.4%)
Chi-square test for association between baseline time-to-antibiotic category and change over time was 16.75, *p*=0.002			

© 2021 Wayne MT et al. *JAMA Network Open.*

eTable 5. Hospital Characteristics by Baseline Time-to-Antibiotic Administration by Tertile

		Tertile 1 (slowest)	Tertile 2	Tertile 3 (fastest)	P for difference by tertile
Mean number of hospitalizations per year during study period, N (%)					
	<2000	15 (34.8)	14 (32.5)	11 (25.0)	0.19
	2000-3999	10 (23.3)	3 (7.0)	11 (25.0)	
	4000-5999	10 (23.3)	10 (23.3)	12 (27.3)	
	6000+	8 (18.6)	16 (37.2)	10 (22.7)	
Mean number of sepsis hospitalizations per year during study period, N (%)					
	<50	10 (23.3)	9 (20.9)	8 (18.2)	0.69
	50-99	10 (23.3)	6 (14.0)	9 (20.4)	
	100-199	13 (30.2)	11 (25.6)	15 (34.1)	
	200+	10 (23.2)	17 (39.5)	12 (27.3)	
Region, N (%)					
	Northeast	7 (16.3)	6 (14.0)	8 (18.2)	0.67
	Midwest	8 (18.6)	13 (30.2)	13 (29.5)	
	South	21 (48.8)	14 (32.6)	15 (34.1)	
	West	7 (16.3)	10 (23.2)	8 (18.2)	
Teaching hospital, N (%)					
	Yes	18 (41.9)	22 (51.2)	21 (47.7)	0.68
	No	25 (58.1)	21 (48.8)	23 (52.3)	
Location, N (%)					
	Urban	35 (81.4)	39 (90.7)	39 (88.6)	0.41
	Rural	8 (18.6)	4 (9.3)	5 (11.4)	

© 2021 Wayne MT et al. *JAMA Network Open.*

eTable 6. Hospital Characteristics by Change in Time-to-Antibiotic Administration Over Time Tertile

	Tertile 1 (greatest change)	Tertile 1 (greatest change)	Tertile 1 (greatest change)	P for difference by tertile
Mean number of hospitalizations per year during study period, N (%)				
<2000	8 (18.2)	13 (30.2)	19 (44.2)	0.02
2000-3999	11 (25.0)	4 (9.3)	9 (20.9)	
4000-5999	16 (36.4)	10 (23.3)	6 (14.0)	
6000+	9 (20.4)	16 (37.2)	9 (20.9)	
Mean number of sepsis hospitalizations per year during study period, N (%)				
<50	5 (11.4)	8 (18.6)	14 (32.6)	0.04
50-99	6 (13.6)	7 (16.2)	12 (27.9)	
100-199	18 (40.9)	14 (32.6)	7 (16.3)	
200+	15 (34.1)	14 (32.6)	10 (23.2)	
Region, N (%)				
Northeast	5 (11.4)	6 (14.0)	10 (23.3)	0.55
Midwest	10 (22.7)	11 (25.6)	13 (30.2)	
South	19 (43.2)	16 (37.2)	15 (34.9)	
West	10 (22.7)	10 (23.2)	5 (11.6)	
Teaching hospital, N (%)				
Yes	23 (52.3)	22 (51.2)	16 (37.2)	0.29
No	21 (47.7)	21 (48.8)	27 (62.8)	
Location, N (%)				
Urban	39 (88.6)	39 (90.7)	35 (81.4)	0.41
Rural	5 (11.4)	4 (9.3)	8 (18.6)	

© 2021 Wayne MT et al. *JAMA Network Open.*

eTable 7. Time-to-Antibiotics Over Time by Sepsis Subgroups

	Early 2013-2014 (n=34,985)	Middle 2015-2016 (n=35,731)	Late 2017-2018 (n=40,669)	*p* for difference in temporal trend between groups*
Subgroups by presenting temperature				
Hyperthermic (>38 Celsius)	3.3 (2.0, 5.5)	2.9 (1.8, 4.6)	2.6 (1.7, 4.1)	0.047
Hypothermic (<36 Celsius)	5.1 (3.0, 7.6)	4.8 (2.8, 7.3)	4.1 (2.5, 6.7)	0.623
Normothermic (≥36 and ≤38 Celsius)	4.7 (2.8, 7.3)	4.2 (2.6, 6.9)	3.8 (2.4, 6.2)	Ref.
Subgroups by presenting blood pressure				
Hypotensive (SBP<90mmHg)	3.9 (2.3, 6.5)	3.5 (2.2, 5.9)	3.0 (1.9, 5.2)	0.463
Normotensive (SBP 90+ mmHg)	4.5 (2.7, 7.1)	4.0 (2.5, 6.6)	3.6 (2.3, 6.0)	

**p* calculated using linear regression models that tested for an interaction between subgroup (temperature, and separately blood pressure) and calendar year.
SBP: systolic blood pressure

© 2021 Wayne MT et al. *JAMA Network Open.*

eReferences

1. Division of Healthcare Quality Promotion. Centers for Disease Control and Prevention. Hospital Toolkit for Adult Sepsis Surveillance. 2018;(May):1-28. https://www.cdc.gov/sepsis/pdfs/Sepsis-Surveillance-Toolkit-Aug-2018_508.pdf

2. Taylor SP, Anderson WE, Beam K, Taylor B, Ellerman J, Kowalkowski MA. The Association Between Antibiotic Delay Intervals and Hospital Mortality Among Patients Treated in the Emergency Department for Suspected Sepsis. *Crit Care Med*. Published online February 15, 2021. doi:10.1097/CCM.0000000000004863

3. Wang XQ, Vincent BM, Wiitala WL, et al. Veterans Affairs patient database (VAPD 2014-2017): Building nationwide granular data for clinical discovery. *BMC Med Res Methodol*. 2019;19(1):1-9. doi:10.1186/s12874-019-0740-x

© 2021 Wayne MT et al. *JAMA Network Open.*
